# Characteristics of Metachronous Gastric Tumors after Endoscopic Submucosal Dissection for Gastric Intraepithelial Neoplasms

**DOI:** 10.1155/2014/863595

**Published:** 2014-02-11

**Authors:** Tomoyuki Boda, Masanori Ito, Shiro Oka, Yoko Kitamura, Norifumi Numata, Yoji Sanomura, Taiji Matsuo, Shinji Tanaka, Masaharu Yoshihara, Koji Arihiro, Kazuaki Chayama

**Affiliations:** ^1^Department of Gastroenterology and Metabolism, Hiroshima University, 1-2-3 Kasumi, Minami-Ku, Hiroshima 734-8551, Japan; ^2^Department of Endoscopy, Hiroshima University Hospital, 1-2-3 Kasumi, Minami-Ku, Hiroshima 734-8551, Japan; ^3^Health Service Center, Hiroshima University, 1-7-1 Kagamiyama, Higashi-Hiroshima 739-8514, Japan; ^4^Department of Pathology, Hiroshima University Hospital, 1-2-3 Kasumi, Minami-Ku, Hiroshima 734-8551, Japan

## Abstract

*Background.* Recently, endoscopic submucosal dissection (ESD) has become a standard treatment method for early gastric cancer and concurrent stomach preservation. However, metachronous recurrences have become a major problem. We evaluated the incidence and clinicopathologic features of and examined the risk factors for metachronous gastric tumors. *Methods.* A total of 357 patients who underwent ESD for gastric tumors (245 early gastric cancers and 112 adenomas) and were followed up for more than 12 months without recurrence within the first 12 months were enrolled. We investigated the incidence and clinicopathologic features of metachronous tumors after ESD. We also analyzed the potential risk factors for metachronous tumors using the Kaplan-Meier method and Cox's proportional hazards model. *Results.* The annual incidence of metachronous tumors after ESD was 2.4%. The median period until discovery after initial ESD was 26.0 months, and the median observation period was 52.6 months. Male patients developed metachronous tumors more frequently (*P* = 0.04), and the hazard ratio of female to male patients was 0.36 (95% confidence interval: 0.11–0.89). *Conclusions.* Patients with a previous history of gastric tumors have a high risk of subsequent gastric tumor development and male patients should be carefully followed up after ESD for gastric tumor.

## 1. Introduction

Gastric cancer is the second most frequent cause of cancer death, and the incidence of gastric cancer among developed countries is the highest in Japan [1]. A number of epidemiological studies have indicated that *Helicobacter pylori *(*H. pylori*) infection is significantly related to gastric cancer development [2–4]. Approximately 10–20% of gastric cancer patients develop multiple synchronous and metachronous cancers [5–8]. To detect early gastric cancer (EGC) after treatment, surveillance procedures should be carefully adhered to.

In recent years, endoscopic submucosal dissection (ESD) for EGC has been widely performed in Japan. With this method, stomach preservation and maintenance of the patients' quality of life are possible [9–12]. However, this approach has been associated with an increase in the risk of gastric cancer recurrence, especially metachronous multiple cancers. The cumulative 3-year incidence of metachronous multiple gastric cancer after partial gastrectomy for EGC was reported to be 1.9% [13]. Previous studies also reported that the annual incidence of metachronous multiple gastric cancer after ESD for EGC was 2.6–3.5% [7, 14, 15]. However, the median observation periods of these studies are short (less than 3 years) and there is no study using gastric tumor including adenoma, which generally becomes indication for ESD in Japan, because the pathological finding after ESD occasionally shows adenocarcinoma even though preoperative biopsy showed adenoma [16].

In the present study, we evaluated the incidence and clinicopathologic features of metachronous multiple tumors that developed during long-term observation and investigated whether we could predict the occurrence of such tumors on the basis of the patient and tumor features during initial ESD.

## 2. Materials and Methods

### 2.1. Patients

We enrolled 1,087 consecutive patients with gastric tumors (766 EGCs and 321 adenomas) who underwent ESD at Hiroshima University Hospital between April 2002 and May 2010. We excluded patients with 28 previous gastric surgical histories, 11 local gastric tumor recurrences, 6 gastric mucosa-associated lymphoid tissue lymphoma, 3 Barrett's adenocarcinoma, and 611 patients who had not been followed-up for more than 12 months. Sixty patients who underwent gastric surgery after ESD and 11 patients who underwent *H. pylori* eradication therapy were also excluded. A final total of 357 patients (273 male, 84 female; mean age: 67.4 years) were enrolled in this study, including 245 EGC patients and 112 adenoma patients. Three hundred and thirty-five patients (94%) were resected as curative resection according to the Japanese gastric cancer treatment guidelines [17] and others were observed without additional surgical resection. The median observation period was 52.6 months (range: 12.2–113.4 months). Three hundred and twelve patients (88%) were followed up by annual endoscopic examination in our hospital. We defined a metachronous tumor as a new tumor that developed in another region at least 12 months after ESD.

The protocol was approved by the Ethics Committee of Hiroshima University Hospital (number 669).

### 2.2. Evaluation of Clinicopathologic Features

We investigated the incidence of metachronous tumors in 357 patients using the Kaplan-Meier method and retrospectively investigated the clinicopathologic features associated with metachronous tumors, including patient age and gender, tumor size, location, gross type, extension of gastric mucosal atrophy, presence of synchronous multiple tumors, histology, and depth. We also evaluated the outcomes of metachronous tumors after ESD.

In patients with synchronous multiple tumors, we chose as the main lesion a tumor that had the highest malignant potential as determined by a malignancy, diffuse type, or increased size or depth. Tumor location and macroscopic types of gastric tumors were classified according to the Japanese Classification of Gastric Carcinoma (JCGC) [18]. In this study, type 0-I (protruded) and type 0-IIa (superficial elevated) were grouped together as “elevated,” while type 0-IIc (superficial depressed) and type 0-IIa+IIc (elevated with central depression) were grouped together as “depressed.” Endoscopic evaluations of atrophic gastritis were determined according to the criteria of the Kimura and Takemoto classification [19]. The pathological diagnosis of each tumor was also judged according to the JCGC criteria [18]. In this study, we included adenoma among the intestinal-type tumors.

### 2.3. Evaluation of Serum Markers

We evaluated the levels of serum gastrin (Gastrin RIA Kit II; Dainabot Co., Ltd., Osaka, Japan) and serum pepsinogen (LZ test; Eiken Chemical Co., Ltd., Tokyo, Japan) instead of performing histological evaluation of the gastric mucosa. We could evaluate fasting serum gastrin and pepsinogen levels in 281 of the 357 patients.

### 2.4. Statistics

The cumulative incidence of metachronous gastric tumors was evaluated using the Kaplan-Meier method. To analyze potential risk factors for metachronous tumors, we performed univariate analysis using the Kaplan-Meier method, log-rank test, and Cox's proportional hazards modeling. A *P* value of <0.05 was considered significant. The JMP statistical software package (SAS Institute Inc., Cary, NC, USA) was used for all calculations. 

## 3. Results

### 3.1. Kaplan-Meier Analysis of the Cumulative Incidence of Metachronous Gastric Tumors

**Figure 1:**
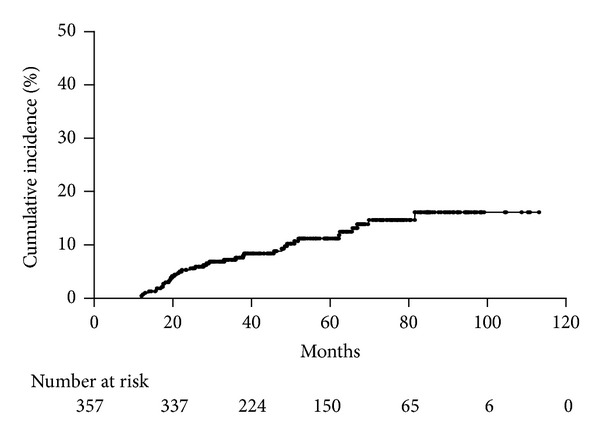
Kaplan-Meier curve of the cumulative incidence of metachronous tumors after ESD for gastric tumors.

We investigated the incidence of metachronous gastric tumors after ESD in 357 patients with gastric tumors using the Kaplan-Meier method (Figure 1). Thirty-nine patients developed metachronous tumors (24 EGCs and 15 adenomas), and the median period until discovery after initial ESD was 26.0 months (range: 12.2–81.8 months). According to the investigation of initial/metachronous tumor, 5 patients had adenoma/adenoma, 2 patients had adenoma/adenocarcinoma, 10 patients had adenocarcinoma/adenoma, and 22 patients had adenocarcinoma/adenocarcinoma, respectively. The cumulative incidence curve of metachronous gastric tumors revealed a gradual increase and an incidence of 2.4% per year. When we excluded cases in which the initial or second tumors were adenoma from the 357 patients, the incidence of metachronous EGC was 2.0% per year (*n* = 236, data not shown). There was no difference in the incidence of metachronous gastric tumor between adenoma and adenocarcinoma of initial treatment.

### 3.2. Clinicopathologic Characteristics of Metachronous Gastric Tumors after ESD

We investigated the clinicopathologic characteristics of the tumors and patients at the time of second tumor discovery in the above-mentioned 39 patients who developed metachronous tumors (Table 1). The average age was 70.3 years, and 35 (90%) of the patients were male. The average tumor size was 11.1 mm (range: 3–20 mm) in diameter. Of 39 lesions, 10 (26%), 12 (31%), and 17 (44%) lesions developed in the upper, middle, and lower third of the stomach, respectively. When we compared the second tumors and initial tumors with regard to development location, the second tumors more frequently developed in the upper third of the stomach (*P* = 0.0002). Eighteen (46%) lesions were diagnosed as elevated type and the others were of depressed type. Almost all patients had severe gastric mucosal atrophy, which is termed as open-type according to the Kimura-Takemoto classification. Only 3 (8%) patients had developed multiple tumors at the time of second tumor detection. According to the pathological evaluation, 37 (95%) patients developed intestinal-type tumors and the others developed diffuse-type tumors. Five cases (13%) developed submucosal invasive gastric cancers. No advanced gastric cancers occurred. All intramucosal tumors were curatively resected by ESD. Four patients with submucosal gastric cancers underwent additional resection of the stomach, and 1 patient was followed up without surgery. There were no gastric cancer deaths during follow-up period.

### 3.3. Analysis of Risk Factors for Metachronous Gastric Tumors after ESD

**Figure 2:**
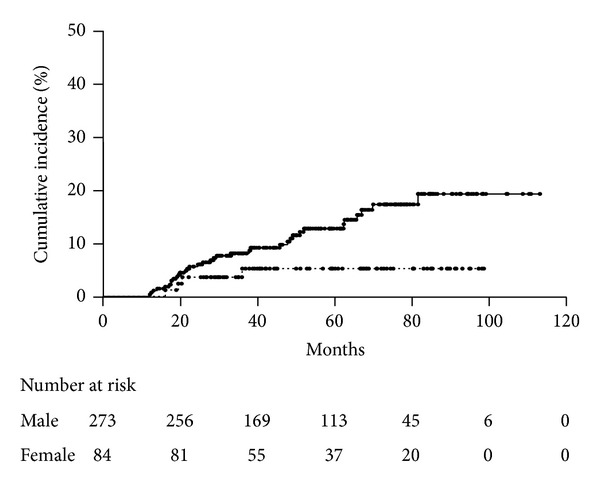
Cumulative incidence of metachronous tumors after ESD in male and female patients. The incidence of metachronous tumors was significantly greater among male patients (solid line) than among female patients (dotted line; *P* = 0.04).

According to the univariate analysis performed using the Kaplan-Meier method and log-rank test, only gender significantly affected the incidence of metachronous tumors. The incidence of metachronous tumors was greater among male patients than among female patients (*P* = 0.04, Figure 2). As shown in Table 2, the hazard ratio of female to male patients was 0.36 (95% confidence interval: 0.11–0.89), and no other factors affected the incidence in the univariate analysis according to Cox's proportional hazards model. 

## 4. Discussion

In Japan, ESD has been standardized as a local treatment for EGC with no risk of lymph node (LN) metastasis. According to the Japanese gastric cancer treatment guidelines [17], ESD is indicated as a standard treatment for differentiated-type adenocarcinomas without ulcerative findings (UL(+)), with a depth of invasion clinically diagnosed as T1a and a diameter of ≤2 cm (absolute indication). Tumors that are clinically diagnosed as T1a and are (a) of the differentiated type, UL(−), but >2 cm in diameter; (b) of the differentiated type, UL(+), and ≤3 cm in diameter; or (c) of the undifferentiated type, UL(−), and ≤2 cm in diameter have a very low possibility of LN metastasis, and ESD for these tumors is regarded as an investigational treatment (expanded indication). Additionally, resection of differentiated-type adenocarcinomas with submucosal invasion of <500 *μ*m and a diameter of ≤3 cm is considered curative. Some reports have supported the validity of these indications [20–22]. Furthermore, risk factors for LN metastasis of submucosal invasive gastric cancer or undifferentiated type EGC have been reported [23–29]. We have been able to perform resection in difficult-to-treat cases such as those with ulceration because of advances in the ESD technique and device [30, 31]. ESD may have a potential that the criteria for curative endoscopic resection (ER) is increasingly expanded in the future. It is commonly known that gastric cancers often recur metachronously, and the risk of metachronous multiple tumors after ESD is thought to be higher than that after gastrectomy [7, 13–15].

Our data revealed that the annual incidence of metachronous gastric tumors was 2.4%, which is almost equal to the previously reported incidence [7, 14, 15]. The median interval period to the detection of a second tumor after initial ESD was 26.0 months (range: 12.2–81.8 months), and Kaplan-Meier curve seemed to reach a plateau after 80 months. Kobayashi et al. [32] reported that the median interval between the discovery of metachronous cancer and the initial ER was 3.2 years in patients who were followed up for 3.0 to 19.6 years (median: 5.0 years), and no metachronous cancers were detected in patients who were followed up for more than 10 years. These data suggest that metachronous gastric tumors may develop around 3 years after ESD and that the incidence may gradually decrease after ESD. However, it is necessary to investigate more cases because of too few patients followed up during long term. Since the annual incidence of gastric cancer in *H. pylori*-positive patients was reported to be 0.38–0.5% [4, 33], after ESD, patients have a higher risk of developing gastric cancer.

We investigated the clinicopathologic characteristics of metachronous gastric tumors and revealed that second lesions tended to develop in the upper third of the stomach. Kato et al. [15] reported that many synchronous gastric cancers after ESD that had been missed by the preoperative endoscopic examination were located in the upper third of the stomach. Since it is difficult to detect tumors in this region, we might be able to detect them by more frequent and careful endoscopic examinations after ESD.

Our data showed that only gender significantly affected the metachronous tumor incidence. Some reports indicated that male patients more frequently developed metachronous gastric cancer after surgery or ER [13, 15, 32]. It is commonly known that the incidence of gastric cancer is higher in male than in female. It has also been reported that differences in smoking rates and salt intake between male and female affect the incidence [34, 35]. Patient age and the presence of synchronous multiple gastric cancers at the time of the initial ER have been reported to significantly affect the incidence of metachronous gastric cancer [36], and antral atrophy was significantly associated with incidence in a previous multivariate analysis [14]. However, these factors did not significantly affect the results of this study. The fact that patients with synchronous tumors are susceptible to metachronous tumors implies that gene mutations or gastric mucosal conditions may be causes of metachronous tumors. A few reports indicated that microsatellite instability (MSI) was a factor that affected the development of both synchronous and metachronous multiple gastric cancers, and the frequency of MSI was found to be significantly higher in patients with metachronous gastric cancers than in those with single gastric cancers [37, 38]. Although we could not evaluate the histological condition of the gastric mucosa, we investigated serum gastrin and pepsinogen levels instead. Serum gastrin levels of patients with severe gastric mucosal atrophy are higher than those of patients with mild or no gastric mucosal atrophy because severe atrophy reduces the secretion of gastric acid [39, 40]. Serum levels of pepsinogen (PG) I and PG II and the PG I/II ratio vary according to gastric mucosal atrophy and inflammation [40–42]. Cases with PG I ≤ 70 ng/mL and PG I/II ≤ 3 were regarded as PG positive, indicative of gastric mucosal atrophy [41]. It was thought that gastric mucosal condition seldom affected the metachronous tumor incidence because serum gastrin and pepsinogen levels did not affect the incidence. In this study, we excluded patients who received *H. pylori* eradication therapy to avoid the influence of *H. pylori* eradication on the development of metachronous tumor. Eradication of *H. pylori *infection was reported to reduce the risk of gastric cancer development [33, 43]. Recently, an open-label, randomized controlled trial showed that *H. pylori* eradication prevented the development of metachronous cancer after ER for EGC patients during a 3-year follow-up period [7], and therefore, eradication therapy is recommended after ER for EGC in Japan [44]. As a result, metachronous gastric cancer detection after *H. pylori *eradication will increase. Furthermore, some reports suggested that macroscopic/biological features of gastric tumors could change after *H. pylori* eradication [45–47]. In the near future, it will be necessary to investigate predictive factors of metachronous gastric tumors after ESD for gastric tumors in patients who have undergone *H. pylori *eradication.

## 5. Conclusions

Patients with a previous history of gastric tumors have an increased risk of subsequent gastric tumor development and male patients should be carefully followed up after ESD for gastric tumor.

## Figures and Tables

**Table 1:** Clinicopathologic characteristics associated with metachronous gastric tumors.

Factors	No. of patients
Total patients	39
Age (years)	
Mean (range)	70.3 (50–88)
Gender	
Male	35 (90%)
Female	4 (10%)
Tumor size (mm)	
Mean (range)	11.1 (3–20)
Location	
Upper third	10 (26%)
Middle third	12 (31%)
Lower third	17 (44%)
Gross type^†^	
Elevated	18 (46%)
Depressed	21 (54%)
Gastric mucosal atrophy	
Closed	1 (3%)
Open	38 (97%)
Synchronous tumor	
Negative	36 (92%)
Positive	3 (8%)
Histology	
Intestinal	37 (95%)
Diffuse	2 (5%)
Depth	
Mucosa	34 (87%)
Submucosa	5 (13%)

^†^Elevated: 0-I and 0-IIa; depressed: 0-IIc and 0-IIa+IIc.

**Table 2:** Analysis of risk factors for metachronous gastric tumors according to Cox's proportional hazards model.

Factors	No.	Univariate analysis	
		Hazard ratio	95% CI
Age (1-year increment)		1.00	0.97–1.04
Gender			
Male	272	1	
Female	85	0.36	0.11–0.89
Tumor size (increment of 1 mm)		1.01	0.98–1.03
Location			
Upper third	38	1	
Middle third	91	1.68	0.55–7.29
Lower third	228	1.02	0.35–4.32
Gross type			
Elevated	161	1	
Depressed	196	1.07	0.57–2.04
Gastric mucosal atrophy			
Closed	30	1	
Open	327	1.20	0.43–4.99
Synchronous tumor			
Negative	307	1	
Positive	50	1.26	0.47–2.79
Histology			
Intestinal	331	1	
Diffuse	26	0.84	0.20–2.33
Depth			
Mucosa	326	1	
Submucosa	31	1.05	0.25–2.92
Serum gastrin			
≤100 pg/mL	94	1	
>100 pg/mL	187	0.58	0.28–1.21
Serum pepsinogen^†^			
Negative	111	1	
Positive	170	1.23	0.59–2.75

CI: confidence interval.

^†^Serum pepsinogen-positive: PG I ≤ 70 ng/mL and PG I/II ≤ 3.
